# Rising Opportunities in Catalytic Dehydrogenative Polymerization

**DOI:** 10.1021/acscatal.4c08091

**Published:** 2025-02-13

**Authors:** Alejandra Sophia Lozano-Pérez, Pavel Kulyabin, Amit Kumar

**Affiliations:** EaStCHEM, School of Chemistry, University of St. Andrews, North Haugh, St. Andrews KY169ST, U.K.

**Keywords:** Dehydrogenative, Dehydrocoupling, Polymerization, Polyamides, Polyureas, Polyketones, Polyethylenimines

## Abstract

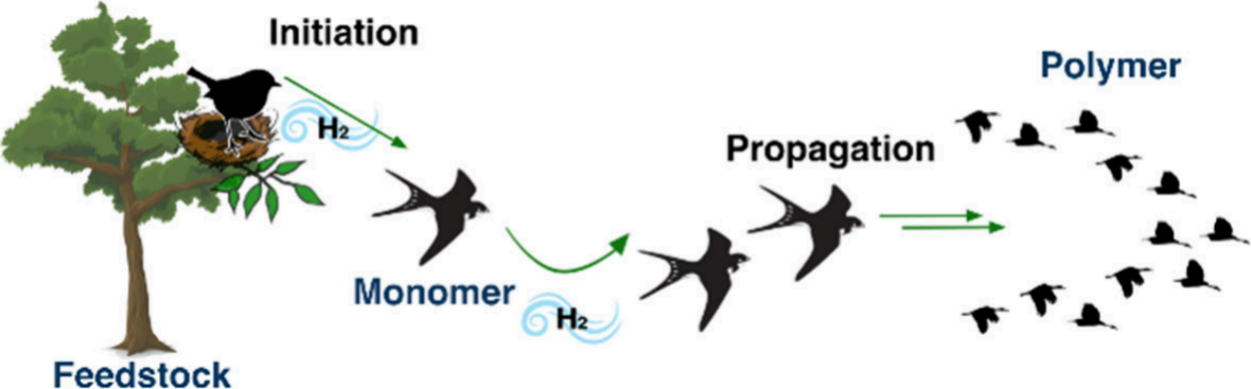

This article gives a perspective on various types of catalytic dehydrogenative polymerization reactions (including organic and main group polymers) while introducing “hydrogen-borrowing polymerization” and “acceptorless dehydrogenative polymerization” to this class. Limitations and future opportunities of each method have been discussed.

## 1 Introduction

**Figure 1:**
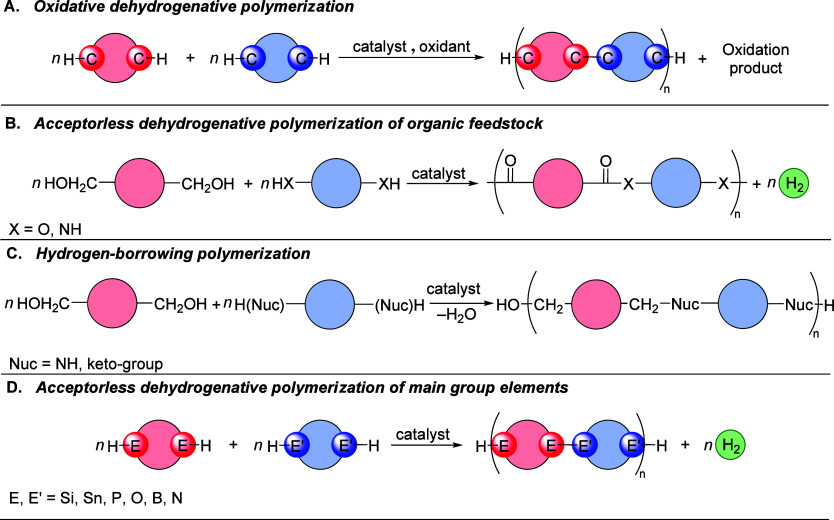
Types of dehydrogenative polymerization.

Dehydrogenative polymerization is a type of condensation polymerization that forms polymers with the extrusion of hydrogen in the presence or absence of an oxidant or acceptor. If the process is achieved in the presence of an oxidant or acceptor, it is called oxidative dehydrogenative polymerization (Figure 1A, often used for polyheteroarenes)^1,2^ whereas if the process is achieved in the absence of an oxidant or acceptor, it can be called acceptorless dehydrogenative polymerization (often used for organic polymers containing carbonyl groups and main group polymers; Figure 1B, D).^1−3^ Often the term “dehydrocoupling” is also used to describe the coupling of main group elements such as amine-boranes, silanes, and phosphine-boranes; however, the process is essentially the same as that of “dehydrogenative coupling”, which is a common term used for the coupling of organic monomers (e.g., arenes, alcohols, and amines).^4,5^ The reason an oxidant is needed in certain cases and not in others depends on both the kinetics and thermodynamics of the process. The dehydrogenative coupling of arenes, e.g., benzene to biphenyl, is thermodynamically uphill and involves high energy for the activation of the sp^2^ C–H bond, needing the assistance of a stoichiometric amount of oxidant or acceptor to drive the process. In contrast, the dehydrogenative coupling of the main group monomer can be thermodynamically downhill (for example, Δ*G* for the dehydrogenative coupling of ammonia borane to form aminoborane is −13.6 kcal mol^–1^ at 298 K), making the process facile without needing any acceptor.^6^ Although the dehydrogenative coupling of alcohols to make esters is also thermodynamically uphill, Δ*G* is not very high and the equilibrium can be pushed toward the product by the continuous removal of hydrogen gas.^7^ We recently demonstrated another related class of polymerization called “hydrogen-borrowing polymerization” (Figure 1C). In this case, the reaction starts with the catalytic dehydrogenation of a starting material such as alcohols, leading to the formation of the active monomers and aldehydes (*initiation step*), followed by condensation with the other starting material such as an amine or a ketone, leading to polymer chain growth (*propagation step*).^8,9^ The intermediates during the polymer chain growth resulting from the condensation process often involve unsaturated bonds such as imines or alkenes that can be hydrogenated using hydrogen (either H_2_ or metal-hydride) produced in the first step (dehydrogenation step). Conceptually, hydrogen-borrowing polymerization can be considered to be somewhere between oxidative and acceptorless dehydrogenative polymerization as H_2_ is both released and consumed in the process. Theoretically, in a fully hydrogen-borrowing process there should not be the release of any H_2_.^10^ It is speculated that the polymerization process terminates by either precipitation of the polymer or deactivation of the catalyst, presumably through thermal degradation.

An advantage of using dehydrogenative polymerization to make known classes of polymers such as polyesters and polyamides in comparison to the conventional routes is that it allows access to alternative feedstocks and polymers of new properties. The release of H_2_ in the case of acceptorless dehydrogenative polymerization can allow the control of polymer chain growth by changing the pressure/concentration of hydrogen gas present in the reaction flask using a number of strategies such as (a) using sealed flasks of different sizes, (b) conducting the reaction under an open flow of inert gas, and (c) pressurizing the reactor with H_2_.^11^ Additionally, there is significant interest in developing polymers that can be made from renewable feedstock and are recyclable. Some feedstocks, for example, alcohols (ethylene glycol, glycerol, ethanol, and methanol) and amines (e.g., priamine) can be made from biomass, and their dehydrogenative coupling can allow the production of fully or semirenewable polymers. Furthermore, some polymers such as polyesters,^12−14^ polyamides,^15,16^ polyurethanes,^17^ and polyureas^18−20^ can also be hydrogenatively depolymerized to produce the feedstock that can be used to make the same polymers.^21^ This presents attractive opportunities for the dehydrogenative polymerization process to enable a circular economy (vide infra).

## 2 Oxidative Dehydrogenative Polymerization

**Figure 2:**
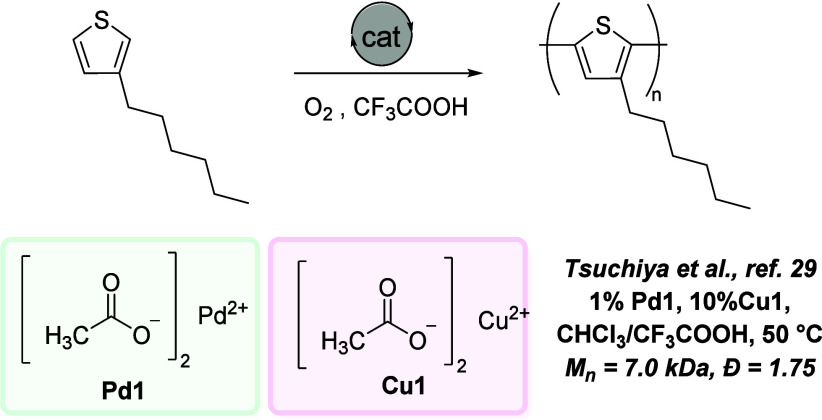
Oxidative polymerization of thiophene derivatives.^29^

**Figure 3:**
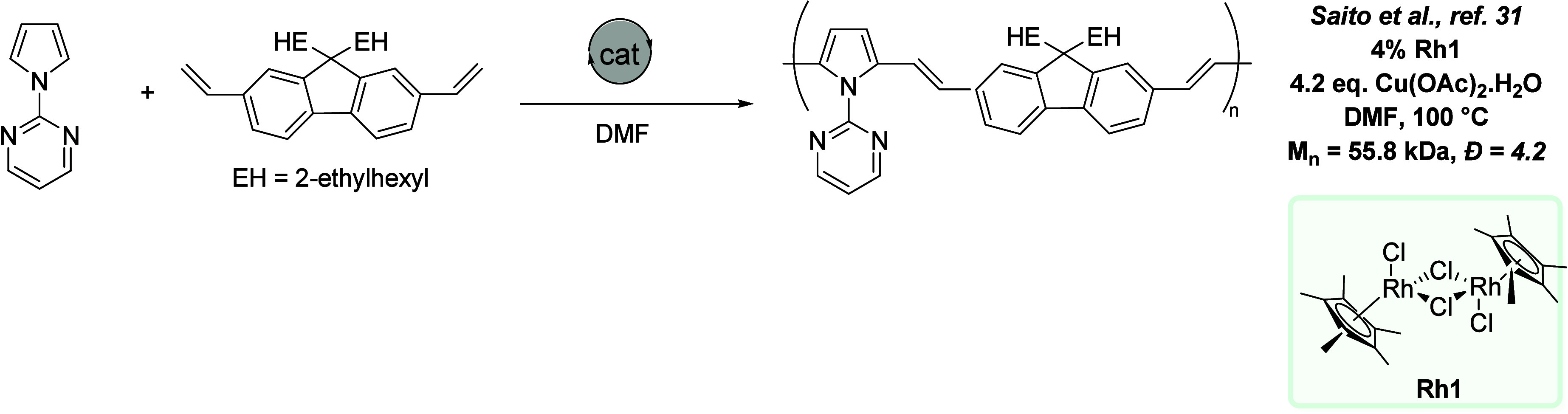
Rh-catalyzed oxidative polymerization of pyrrole-based poly(arylenevinylene)s.^31^

**Figure 4:**
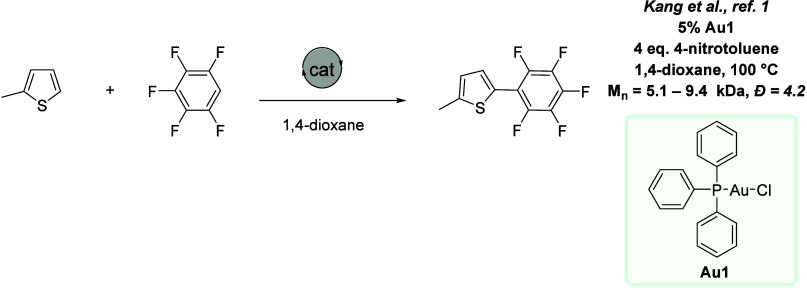
Gold-catalyzed oxidative polymerization.^1^

Oxidative dehydrogenative polymerization has been studied to produce conjugated aromatic polymers via arene–arene or arene–alkene coupling for their applications in organic electronic materials, in particular for the synthesis of donor–acceptor copolymers from the cross-dehydrogenative coupling of different arenes or an arene and alkene. Such polymers are conventionally made from the coupling of haloarenes or haloalkenes via Pd-catalyzed cross-coupling reactions such as Heck^22^ or Suzuki coupling.^23^ The approach of dehydrogenative coupling allows the synthesis of such polymers directly from arenes or alkenes that are cheaper and more abundant feedstocks than halo arenes or haloalkenes.^24^ The area started only a decade ago, and the most common catalysts are based on palladium, such as Pd(OAc)_2_^25−29^ (Figure 2) or Pd(TFA)_2_,^30^ although catalysts based on Rh (Figure 3),^31,32^ Fe,^33^ Cu,^34−36^ and Au (Figure 4) have also been reported for oxidative dehydrogenative polymerization of thiophene- and pyrrole-based monomers.^1,37^ These reactions are achieved using stoichiometric or often excess amounts of oxidants such as K_2_S_2_O_8_ or Ag_2_O, which is problematic from a sustainability perspective as they produce a significant amount of waste that is difficult to treat or regenerate to the same oxidants.^26,38,39^ Often, polar and high-boiling solvents such as DMSO or DMF are used due to their ability to dissolve a wide range of reactants and facilitate reactions at elevated temperatures, which enhances the yield.^40^ Furthermore, their polar aprotic nature allows them to stabilize ionic intermediates without interfering with the reaction mechanism, while their high boiling points prevent solvent evaporation during the process.^41^ Additionally, their compatibility with strong bases further contributes to improved polymer characteristics, making them ideal choices for this type of polymerization.^42^ The use of these solvents, however, creates issues in polymer purification due to the low miscibility with solvents used for precipitation and their high boiling temperatures, which require high energy for removal.^43^ Initial results in this area led to the formation of regiorandom polymers;^44^ however, the selectivity could be improved by tuning the electronics and sterics of the monomers and by using directing groups.^45,46^ There are only a few examples of high-molecular-weight polymers (e.g., >20 k Da), and often the molecular weights of polymer in these cases are lower than 10 k Da. The area is still in its infancy and needs further attention to develop (a) atom-economic catalytic protocols without needing a stoichiometric amount of oxidant or a cheaper/regenerable oxidant and (b) polymers of high regioselectivity and molecular weights.

## 3 Acceptorless Dehydrogenative and Hydrogen-Borrowing Polymerization

### 3.1 Acceptorless Dehydrogenative Polymerization of Organic Feedstock

#### 3.1.1 Dehydrogenative Synthesis of Polyamides and Polyesters

**Figure 5:**
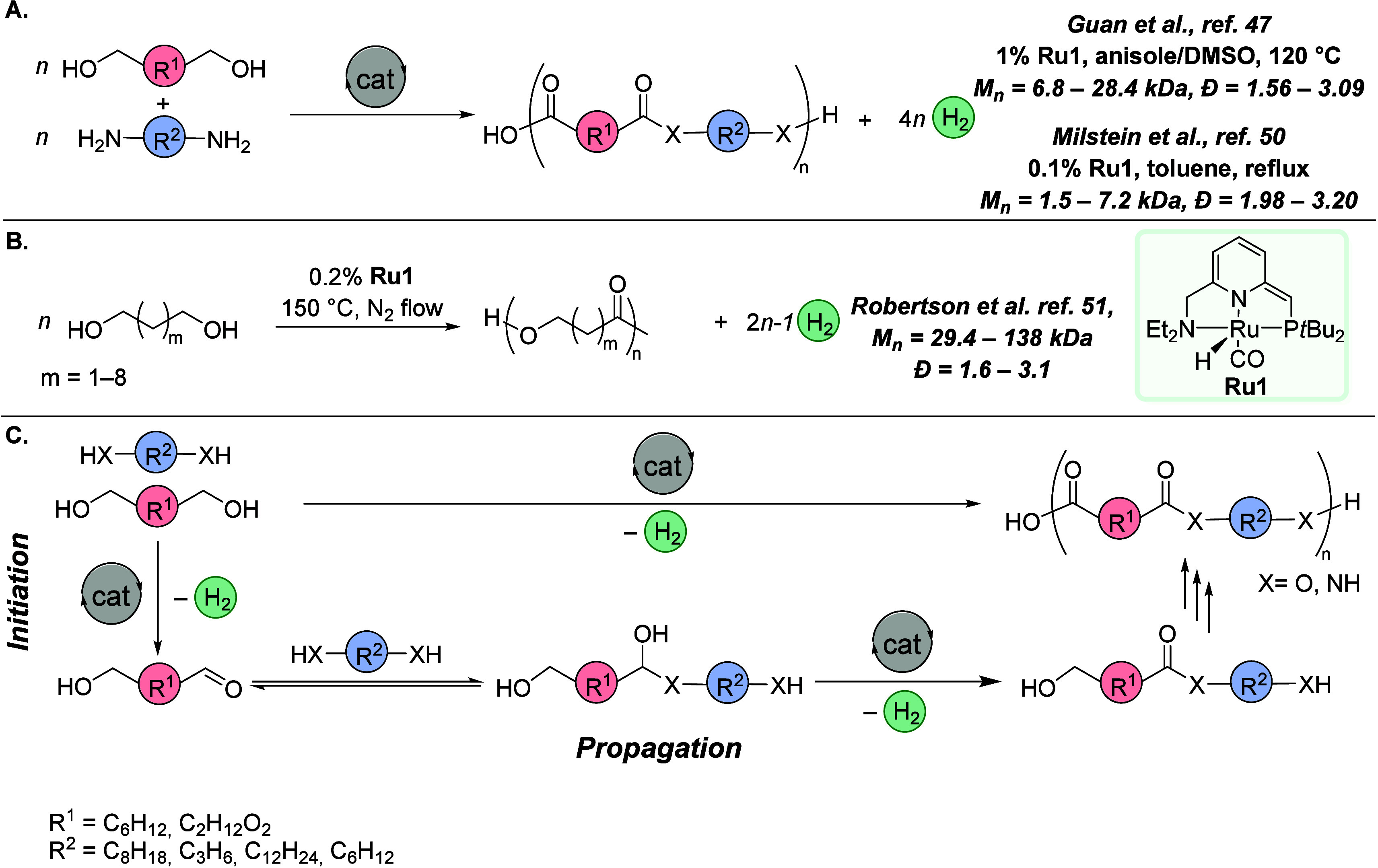
(A) First examples of polyamide syntheses via acceptorless dehydrogenative polymerization. (B) First example of polyester synthesis via acceptorless dehydrogenative polymerization. (C) Mechanism of acceptorless dehydrogenative (co)polymerization of diols and diamines into polyamides and polyesters.

The first example of acceptorless dehydrogenative polymerization of organic feedstock was reported in early 2011 by Zeng and co-workers for the synthesis of polyamides from the dehydrogenative coupling of diols and diamines using the Milstein’s Ru-PNN catalyst (Figure 5, **Ru1**).^47^ The solubility of the resulting polyamides in the reaction medium was a key factor, as extensive hydrogen bonding in the nylon polymer chain could prematurely stop chain growth due to polymer precipitation.^47^ To address this issue, various polar solvents were screened, and a mixture of anisole and DMSO was determined to be optimal, as it improved the solubility of polyamides without decreasing the catalyst’s activity. Using the optimized anisole/DMSO solvent system, a variety of polyamides with *M*_n_ up to 30 kDa were synthesized from a wide range of diols and diamines.^48,49^ Soon after, Milstein and co-workers also reported the synthesis of various polyamides from the dehydrogenative coupling of diols and diamines using the same pincer catalyst.^50^

Around a similar time, in late 2011, Robertson and co-workers used Milstein’s Ru-PNN catalyst (Figure 5, **Ru1**) for the dehydrogenative polymerization of α,ω-diols to produce high-molecular-weight polyesters.^51^ This method was successful for diols with a spacer of six carbons or more, while diols with fewer carbons (1,5-pentanediol or 1,4-butanediol) underwent cyclization into lactones. The authors discovered that performing the polymerization under reduced pressure rather than under a nitrogen purge led to the formation of polyesters of high molecular weight, e.g., up to 145 000 Da. This is consistent with a dehydrogenative mechanism, where the facile release of hydrogen gas can allow a higher concentration of the reactive intermediate (aldehyde), leading to polymer chain growth. This also prevents the hydrogenation of any aldehyde or ester intermediates that would inhibit chain growth. Furthermore, the observation of a regio-irregular polymer chain (based on depressed melting temperature) also suggested that the polymerization proceeds via a dehydrogenative condensation pathway rather than a cyclization and ring-opening polymerization process.

Mechanisms for the dehydrogenative polymerization to make polyesters and polyamides have not been studied in detail; however, the fundamental steps have been suggested to be analogous to the dehydrogenative synthesis of esters and amides (Figure 5C). It has been speculated that the polymerization process starts with the dehydrogenation of diols to form aldehyde-ol or dialdehyde intermediates (*initiation*) that can react with alcohol or amine to form hemiacetal or hemiaminal intermediates, whose subsequent dehydrogenation and continuation of condensation steps could lead to the formation of polyesters or polyamides (*propagation*). We believe that chain *termination* could occur via precipitation when the solubility of the polymer chain reaches the maximum limit in the solvent system. In case the polymerization is conducted in a sealed system, increasing the pressure of H_2_ could also inhibit or stop the dehydrogenation step, leading to chain termination. Although the general mechanism (Figure 5C) shows the possibility of the formation of copolyesters, the dehydrogenative approach has only been utilized for the synthesis of homopolyesters. A possible future direction could look into exploring the formation of copolyesters if one type of diol is preferentially dehydrogenated over another.

#### 3.1.2 Dehydrogenative Synthesis of Polyureas

**Figure 6:**
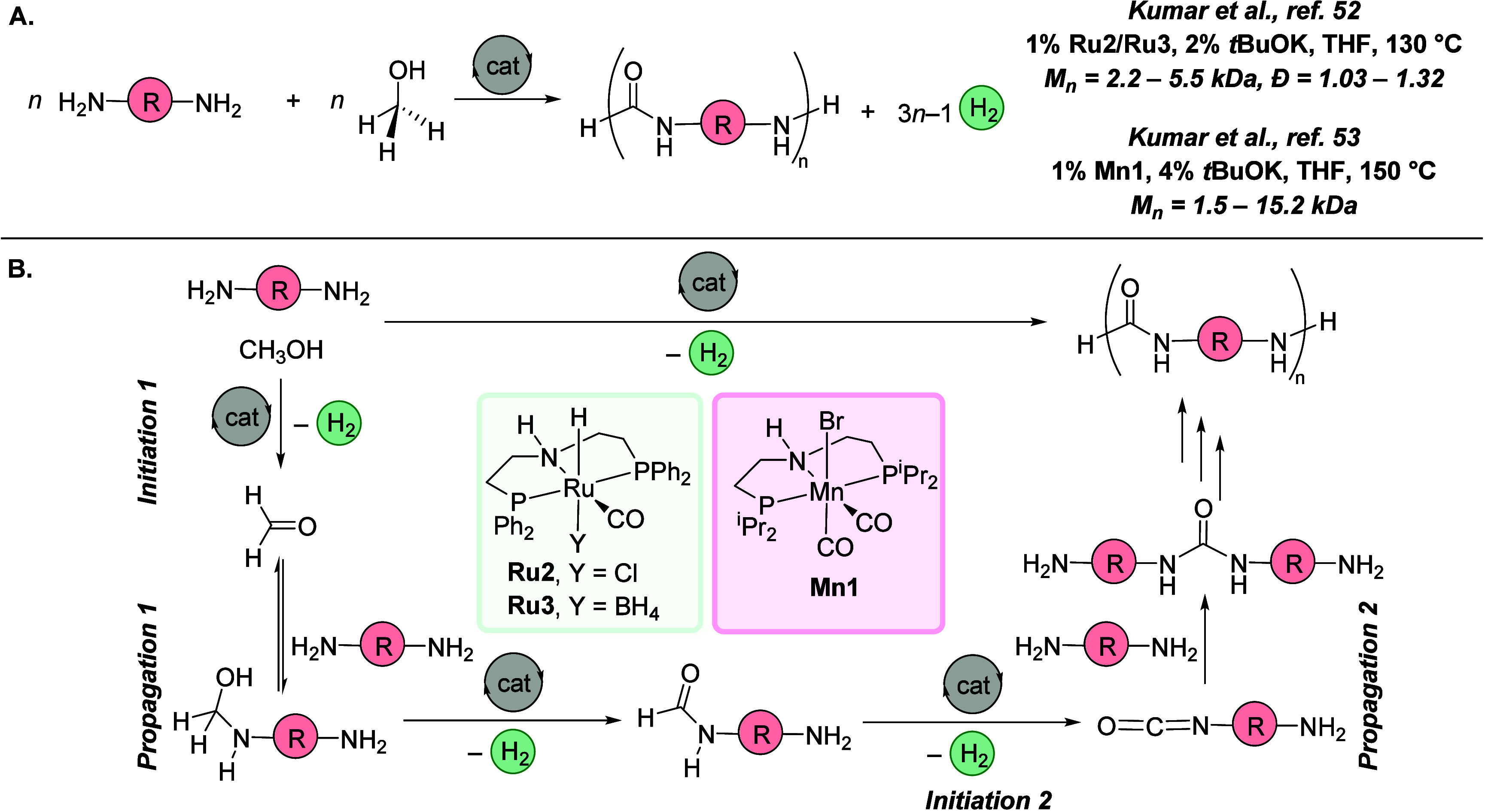
(A) Synthesis of polyureas using acceptorless dehydrogenative copolymerization of diamines and methanol. (B) Proposed mechanism for the dehydrogenative synthesis of polyureas.

After the initial demonstration of the acceptorless dehydrogenative synthesis of polyamides and polyesters in 2011–2012, the area remained inactive for the next 10 years until 2021, when we reported the first synthesis of polyureas via the acceptorless dehydrogenative polymerization of diamines and methanol. The process was first demonstrated using ruthenium^52^ and soon after manganese^53^ pincer catalysts (Figure 6, **Ru1**, **Mn1**). Polymers were obtained in excellent yields (70–95%) and moderate molecular weight with a degree of polymerization in the range of 10–40. This approach was also used for the synthesis of chiral polyureas and the synthesis of the first ^13^C-labeled polyureas using ^13^CH_3_OH.^52^ The mechanism of the polymerization was studied, and the formation of urea derivatives via an isocyanate intermediate was suggested and supported by DFT computation.^53^ As shown in Figure 3, the overall process involves three main steps, each releasing 1 equiv of H_2_. The first step is the transition-metal-catalyzed dehydrogenation of methanol to form formaldehyde (*initiation phase I*). In the second step, formaldehyde reacts with an amine to form a hemiaminal intermediate that becomes dehydrogenated in the presence of a metal catalyst to form a formamide (*propagation phase I*). In the third step, formamide is dehydrogenated to form an isocyanate intermediate (*initiation phase II*) that subsequently reacts with an amine to form a urea derivative (*propagation phase II*). The continuation of these steps leads to the formation of polyurea.

When addressing a specific reaction, for example, coupling of 4,7,10-trioxa-1,13-tridecanediamine and methanol, the outcome of the catalytic reaction (e.g., yield and molecular weight of polymers) was found to depend on a number of conditions such as precatalyst, solvent, and amount of base used (Table 1). Among the studied catalysts,^52^ Ru MACHO (**Ru2**, 1 mol %) showed the best overall yield when using a base loading of 0.04 mmol (2 mol %). When modifying the base loading (entries 1 and 2) or not adding base (entry 4), the yield was found to decrease, suggesting that not only the selection of the catalyst but also the loading of base plays an important role. When comparing **Ru2** and **Ru3**, the yield dropped from 55% to 32%. Complexes **Ru3** and **Ir1** produced similar yields and poor activity relative to that of **Ru2**. Finally, variation in solvent (entries 6–10) was also found to influence the yield and molecular weight of polymers, with THF providing the highest molecular weight among the studied solvents (toluene, DMSO, anisole, and neat).

**Table 1:**
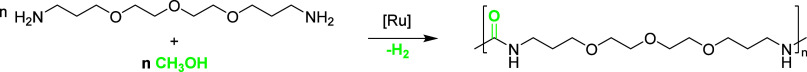
Optimization Table for the Dehydrogenative Coupling of 4,7,10-Trioxa-1,13-tridecanediamine with Methanol^a^,^52^

entry	pre-catalyst	solvent	yield	mol wt (*M*_n_)
1	**Ru2**	toluene	95	4060
2^b^	**Ru2**	toluene	55	2100
3^b^	**Ru3**	toluene	32	1500
4^c^	**Ru3**	toluene	10	
5	**Ir1**	toluene	34	800
6	**Ru2**	anisole	15	2500
7	**Ru2**	DMSO	25	1500
8	**Ru2**	THF	85	4757
9	**Ru2**	neat	25	
10^d^	**Ru2**	toluene	22	

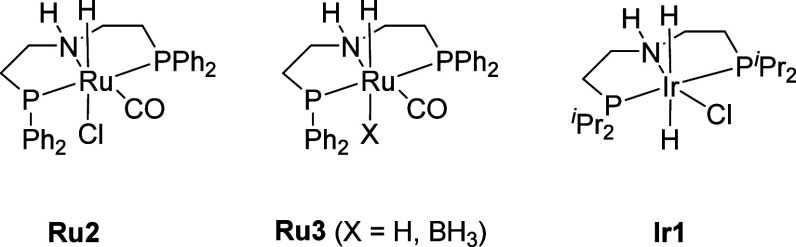

a Catalytic conditions: 4,7,10-trioxa-1,13-tridecanediamine (440 mg, 2 mmol), methanol (0.32 mL, 8 mmol), precatalyst (0.02 mmol), KO^*t*^Bu (4.5 mg, 0.04 mmol), solvent (2 mL), 130 °C, 24 h. *M*_n_ is in Dalton (Da).

b 0.02 mmol KO^*t*^Bu was used.

c No KO^*t*^Bu was used.

d 2 mmol methanol was used.

**Figure 7:**
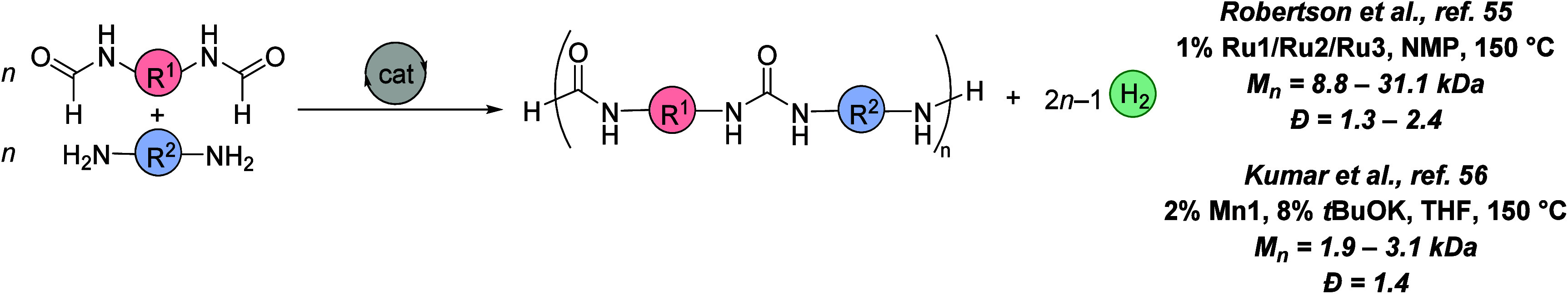
Synthesis of polyureas using acceptorless dehydrogenative copolymerization of diformamides and diamines.

One of the limitations of the aforementioned synthesis of polyureas from diamines and methanol is the issue of functionality. The structure of conventional polyureas involves two functionalities (one coming from aromatic diamines and another one coming from aliphatic diisocyanates), whereas the polyureas made from diamines and methanol contain only one functionality (coming from diamine). The dual functionality is crucial in the physical and mechanical properties of polyureas, as the aromatic functionality provides the rigidity and the aliphatic functionality provides the flexibility to the polymer chain, which are important for the mechanical properties needed for their commercial applications.^54^ To demonstrate the dehydrogenative synthesis of polyureas containing dual functionalities, the dehydrogenative coupling of diformamides and diamines was studied by Robertson using a ruthenium pincer catalyst^55^ and by us using a manganese pincer catalyst (Figure 6, **Mn1**).^56^ Polyureas from aliphatic diformamides and various diamines, including aliphatic and aromatic diamines, were made using ruthenium pincer catalysts. In comparison, the substrate scope was more limited in the case of manganese pincer catalysts (Figure 7).

#### 3.1.3 Dehydrogenative Synthesis of Polyureaurethanes

**Figure 8:**
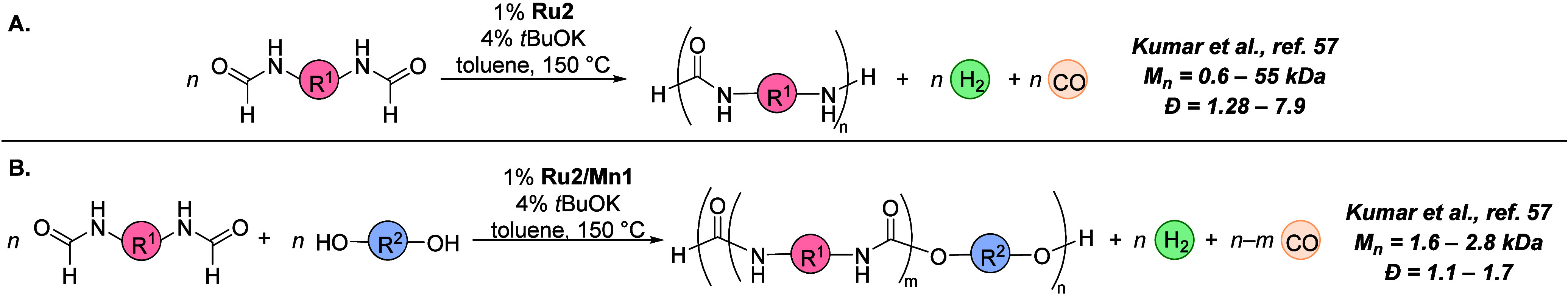
(A) Decarbonylative and dehydrogenative polymerization of diformamides into polyureas. (B) Decarbonylative and dehydrogenative copolymerization of diformamides and alcohols into polyureaurethanes.

**Figure 9:**
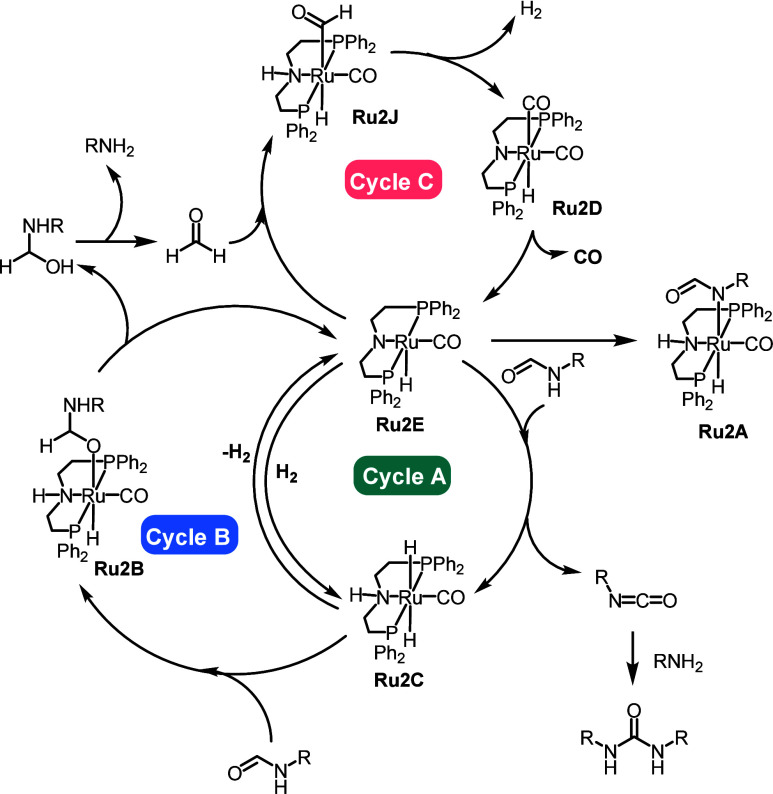
Summary of the mechanism for the dehydrogenation and decarbonylation of formamides catalyzed by the **Ru2E** pincer catalyst. Reproduced from ref (57) with permission. Copyright 2024 Royal Society of Chemistry.

A study to expand this concept to make polyurethanes from the catalytic dehydrogenative coupling of diformamides and diols was also performed (Figure 8).^57^ It was hypothesized that diformamides will dehydrogenate to diisocyanates, which will subsequently react with diols to make polyurethanes. However, the reaction was not straightforward, and a side reaction, i.e., decarbonylation of formamides to amines, was also observed in addition to the dehydrogenation of formamides. This, however, led to the development of a new process where polyureas were directly made from diformamides with the extrusion of CO and H_2_. According to the proposed mechanism (based on experiments and DFT computation), the dehydrogenation of diformamides leads to the formation of diisocyanates that subsequently reacted with diamines formed from the decarbonylation of diformamides to make polyureas. Interestingly, the coupling of diformamides with diols led to the formation of poly(ureaurethanes) as the formation of both urea and carbamate linkages was observed in the catalytic process. The process was studied using ruthenium and manganese MACHO pincer catalysts, and it was found that the selectivity toward carbamate and urea depends on a number of factors, namely catalyst, base (amount and type), and solvent (type, amount). It was also found that ^i^Pr substituents on phosphine in the case of Ru-MACHO (Figure 6, **Ru2**) or Mn-MACHO (Figure 6, **Mn1**) lead to higher selectivity of carbamate derivatives, while increasing the amount of base (KO^t^Bu) and using a ruthenium-based pincer catalyst instead of manganese analogues favor the decarbonylation process. Based on experiments conducted to understand organometallic intermediates and DFT computation, a mechanism involving three catalytic cycles each connected by the activated/deprotonated Ru-MACHO complex (**Ru2E**) was proposed, as mentioned in Figure 9. The process starts with the dehydrogenation of formamide by complex **Ru2E** via an outer-sphere mechanism to form an isocyanate, as shown in cycle A. This results in the formation of the ruthenium–dihydride complex **Ru2C** that enters cycle B to hydrogenate a formamide to form a hemiaminal intermediate, which can decompose to form an amine and formaldehyde. Amine can react with isocyanate produced in cycle A to form a urea derivative, whereas formaldehyde reacts with complex **Ru2E** in cycle C to form a formyl intermediate **Ru2J**. Complex **Ru2J** releases H_2_ via metal–ligand cooperation and forms dicarbonyl complex **Ru2D**, which releases CO to regenerate complex **Ru2E**. According to the DFT computation, the rate-limiting step is the dehydrogenation of formamide to isocyanate with a barrier of Δ*G*^‡^ = 23.3 kcal mol^–1^. The barrier for the decarbonylation was also found to be similar (Δ*G*^423‡^ = 24.3 kcal mol^–1^).

#### 3.1.4 Dehydrogenative Synthesis of Polyurethanes

**Figure 10:**
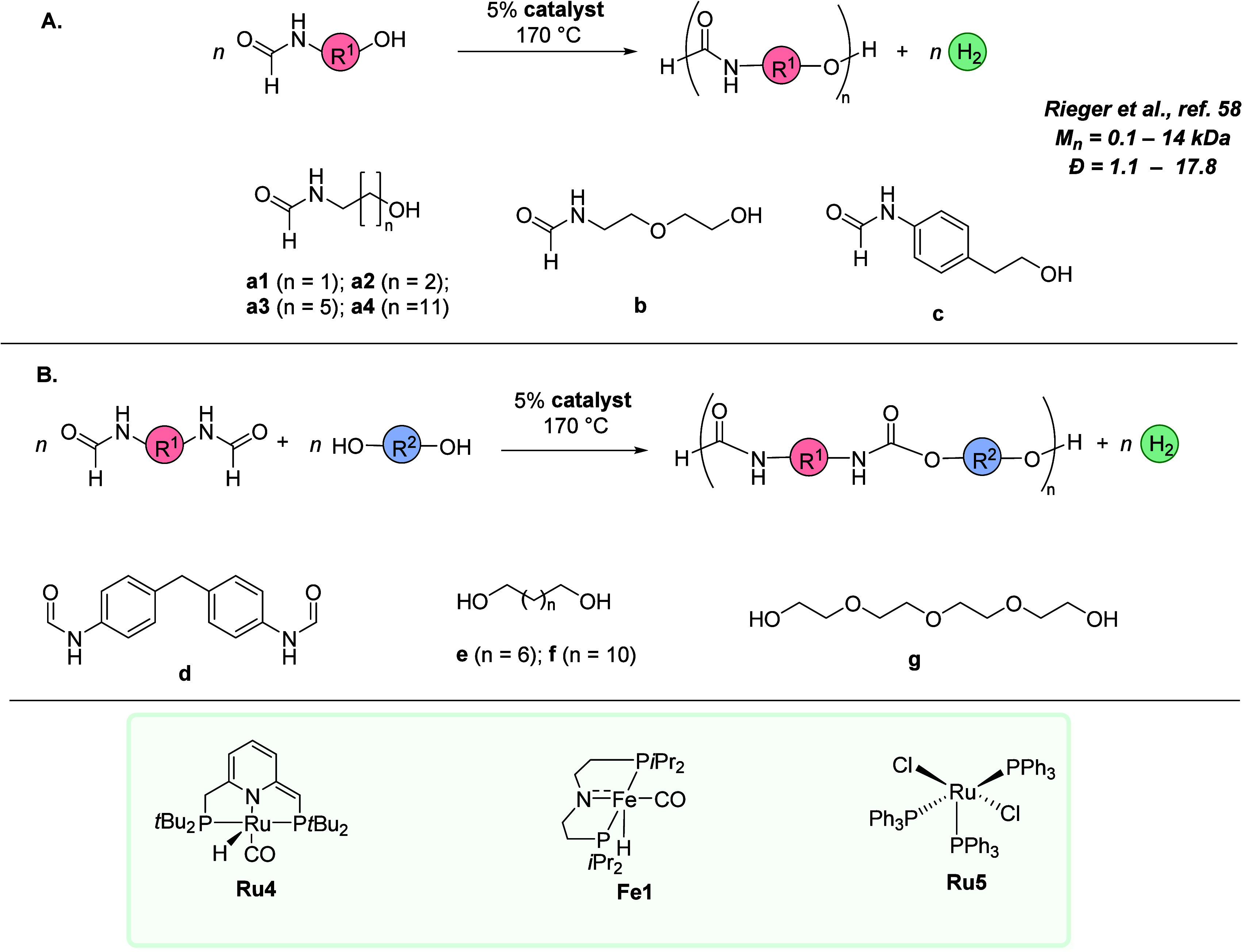
Dehydrogenative synthesis of polyurethanes from (A) formamidol or (B) diformamides and diols.

Recently, the concept of dehydrogenative polymerization has been applied for the synthesis of polyurethanes using ruthenium- and iron-based catalysts (**Ru4**, **Fe1**, and RuCl_2_(PPh_3_)_3_.^58^ These catalysts were previously utilized by Milstein,^59^ Hazari,^60^ and Watanabe^61^ for the synthesis of organic carbamates from the dehydrogenative coupling of formamides and alcohols. Polymerization of a few formamidols (**a1**–**a4**, **b**, and **c**) was studied using these catalysts (5 mol %) at 170 °C in mesitylene at 24 h (Figure 10A). Although the formation of a polyurethane was observed for all the monomers, the degree of polymerization was found to depend on the nature of the monomer as well as the choice of catalyst. The best result was obtained for the formamidol containing aromatic formamide (**c**), which showed 82% conversion of the starting material and *M*_n_ of ∼9000 Da. The formation of precipitate was also observed in these reactions, which through EDX and elemental analysis was proposed to be an oligomer ruthenium network formed via the displacement of PPh_3_ ligands in RuCl_2_(PPh_3_)_3_ by the formed oligomers. To avoid precipitation, more polar solvents were attempted. The highest conversion and molecular weights of polymers were obtained using a 6:1 mixture of anisole/DMSO with LiBr (1 wt %). Using this solvent mixture, a polyurethane of *M*_n_ = 7.6 kDa was obtained from monomer **c**. Additionally, diformamide **d** was also reacted with diols **e**, **f**, and **g**, which led to the formation of polyurethanes in the molecular weight range of 2.7–14.4 kDa (Figure 10B). Based on previous studies,^59−61^ it was suggested that the polymerization proceeds via a dehydrogenative process where the formamide group present in the monomer is dehydrogenated to produce an isocyanate that further reacts with an alcohol group of the monomer to make a carbamate derivative. Subsequent continuation of this process led to the formation of polyurethanes.

### 3.2 Hydrogen-Borrowing Polymerization

**Figure 11:**
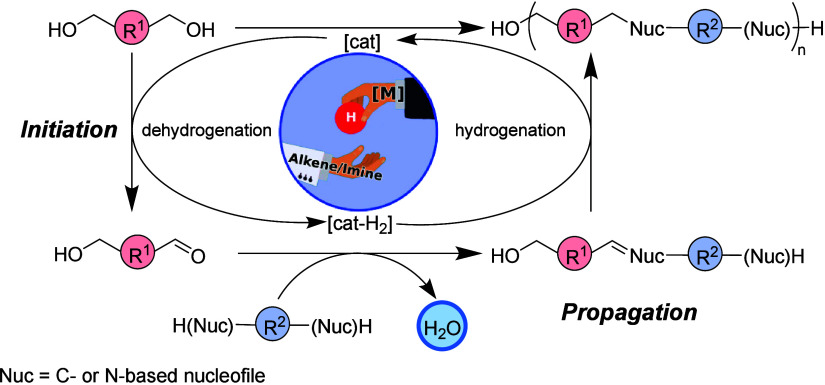
General mechanism of hydrogen-borrowing polymerization.

Hydrogen-borrowing polymerization is a relatively new process recently demonstrated by us that incorporates both dehydrogenation and hydrogenation steps to synthesize polymeric materials. The essential notion of this methodology is that a catalytic metal fragment can temporarily store hydrogen from a hydrogen donor molecule and then later release it to hydrogenate the unsaturated part of the polymer. The strategy typically unfolds in three steps: (i) dehydrogenation, (ii) coupling reaction by condensation, and (iii) hydrogenation. The process begins with a metal-catalyzed dehydrogenation, where a feedstock such as alcohol is converted into a more reactive intermediate aldehyde (*initiation*). This subsequently undergoes condensation with another feedstock to yield unsaturated compounds, such as alkenes or imines (*propagation*), which are later hydrogenated by metal hydrides or H_2_ generated during the initial dehydrogenation (Figure 11).

#### 3.2.1 Synthesis of Polyethylenimines Using Hydrogen-Borrowing Polymerization

We recently demonstrated two new polymerization processes based on the hydrogen-borrowing concept. The first one is the synthesis of branched polyethylenimine derivatives from either ethylene glycol + ethylene diamine feedstock^62^ or directly from ethanolamine feedstock.^63^ Branched polyethylenimines are produced in industry by the ring-opening polymerization of aziridine, which is a highly toxic, volatile, and mutagenic compound. BASF and Nippon Shokubai are the main producers of aziridine. However, due to associated toxicity, they use all of the produced aziridine to make branched polyethylenimines. Using the hydrogen-borrowing polymerization process, we were able to make a derivative of branched polyethylenimine starting from either ethylene glycol + ethylene diamine or directly from ethanolamine by circumventing the need to produce toxic aziridine.

**Figure 12:**
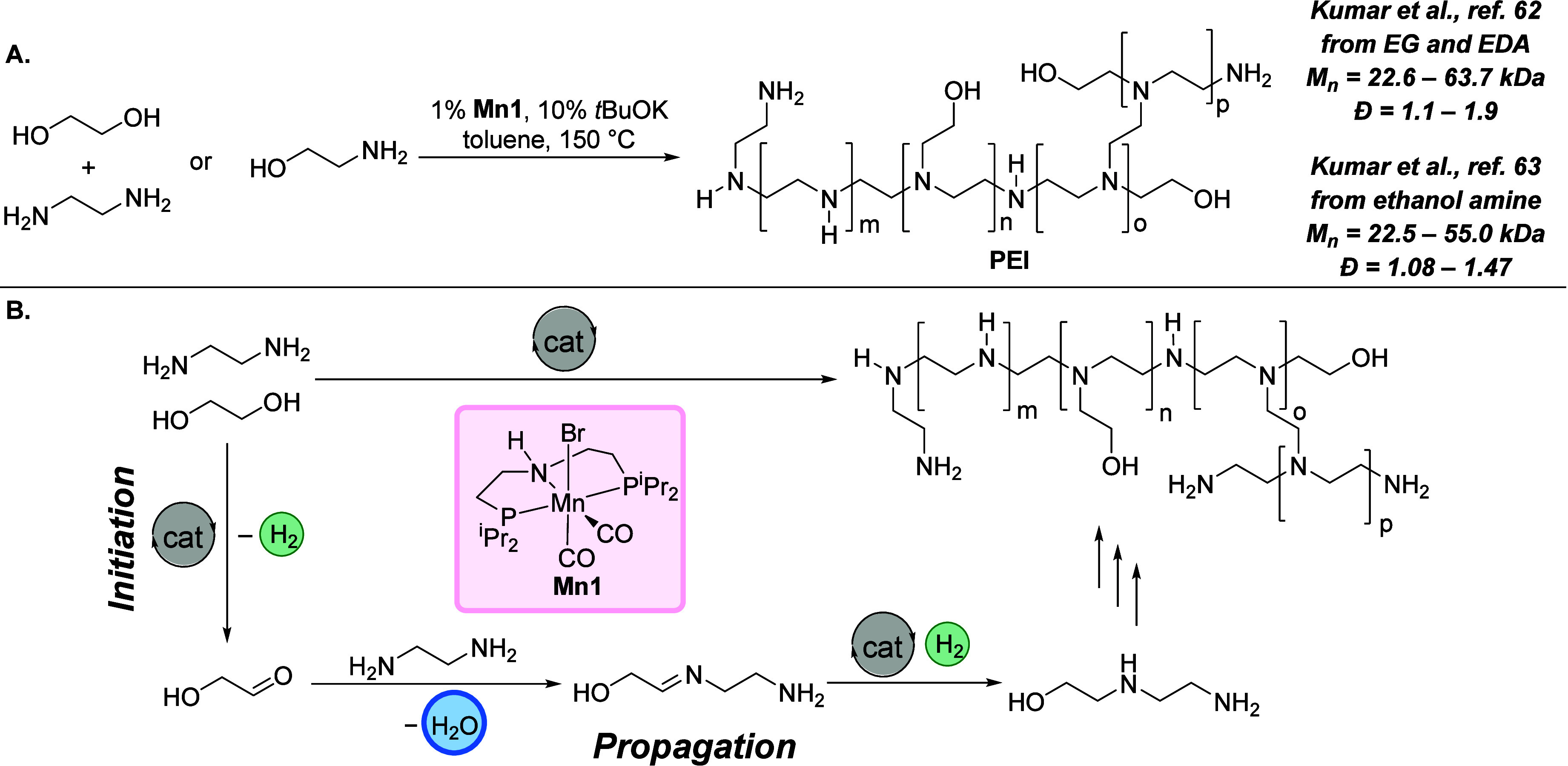
(A) Synthesis of a PEI derivative via hydrogen-borrowing polymerization. PEI = polyethylenimine derivative, EG = ethylene glycol, EDA = ethylenediamine. (B) Proposed mechanism for the formation of PEI via a hydrogen-borrowing process.

A few pincer catalysts based on Mn, Ir, and Ru were used to optimize the conditions for the coupling of ethylene glycol and ethylene diamine, of which manganese pincer complex **Mn1** was found to be the most efficient. Branched polyethylenimine derivatives with high molecular weights (*M*_n_ up to 59 000 g mol^–1^) and narrow *Đ* (1.1–1.4) were formed (Figure 12A).^62^ Interestingly, it was found that the same Mn-MACHO pincer catalyst also works for the synthesis of a similar branched polyethylenimine derivative from the self-coupling of ethanolamine (Figure 9A).^63^ It is noteworthy that in industry aziridine is made from ethanolamine using either an energy-intensive process (e.g., 350–450 °C, by Nippon Shokubai) or concentrated H_2_SO_4_ and NaOH in a two-step process (by BASF) that produces significant waste. Using hydrogen-borrowing polymerization, ethanolamine could be directly converted to a branched polyethylenimine derivative. The analysis by NMR and IR spectroscopy as well as ESI-MS spectrometry showed that the polymer is highly branched and contains some ethoxy linkages in addition to amine linkages. This is different from the commercial polyethylenimines that contain just amine linkages, as all the oxygen atoms are removed during the formation of aziridine. It is likely that the incorporation of oxygen atoms in the polyethylenimines by the virtue of utilized methodology will change some physical and mechanical properties, which need further investigation. In the reported study, it was possible to control the oxygen/nitrogen ratio by doping the ethanolamine feedstock with ethylendiamine.

Mechanisms for both processes are based on hydrogen-borrowing concepts as proposed based on experiments and DFT computation (Figure 12B). The process is initiated by the metal-catalyzed dehydrogenation of alcohol groups present in ethylene glycol or ethanolamine to form either glycolaldehyde or 2-aminoacetaldehyde that reacts with amines to form imines by the elimination of water (chain propagation). Metal-catalyzed hydrogenation of imines leads to the formation of saturated alkylated amines. Computational studies suggested that the dehydrogenation occurs via an outer-sphere mechanism using metal–ligand cooperation with a barrier of Δ*G*^423,15K^ = 10.81 kcal mol^–1^ for ethylene glycol and barrier of Δ*G*^‡^ = 17.7 kcal mol^–1^ for ethanolamine.

#### 3.2.2 Synthesis of Polyketones Using Hydrogen-Borrowing Polymerization

**Figure 13:**
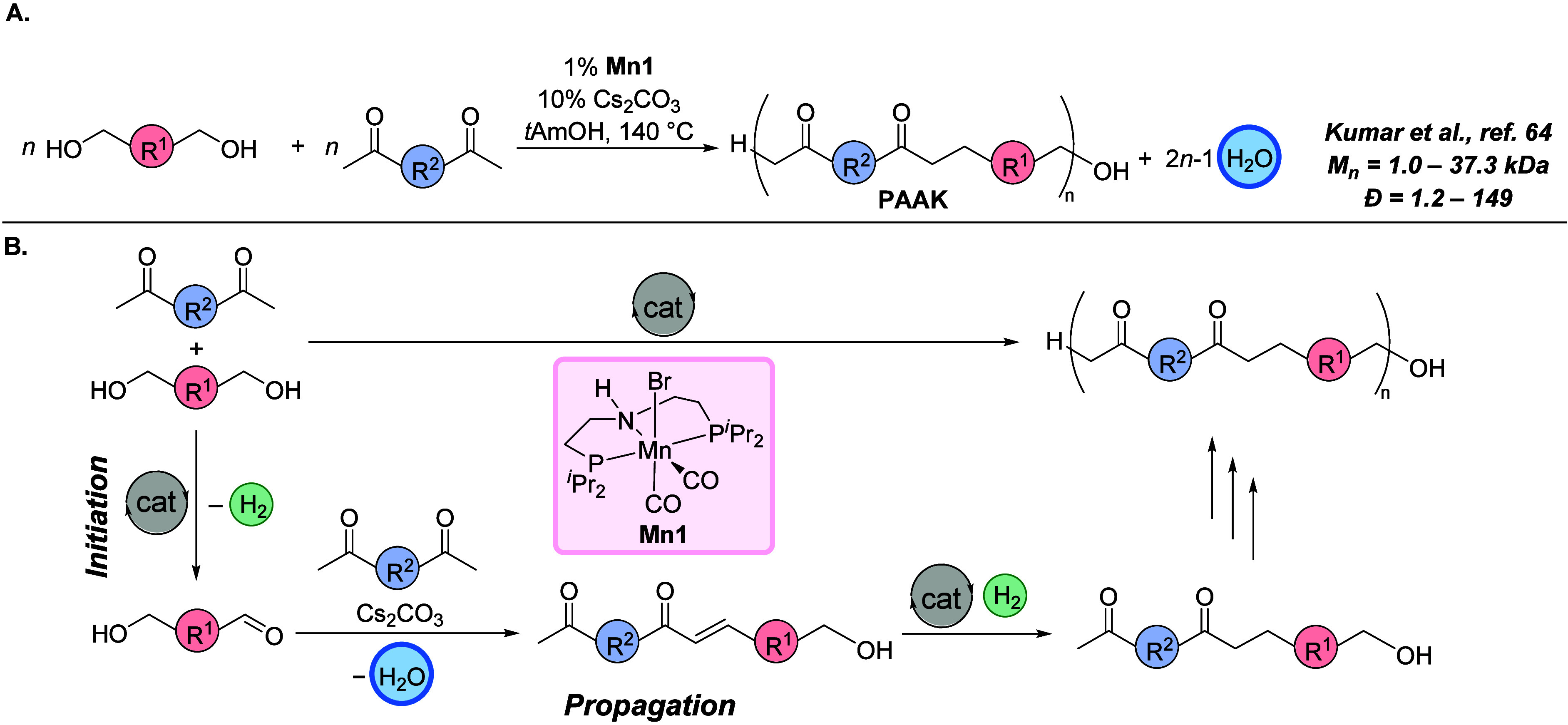
(A) Synthesis of polyarylalkylketones (PAAKs) and (B) proposed mechanism via hydrogen-borrowing polymerization.

Using the concept of hydrogen-borrowing polymerization, we have introduced the synthesis of a new class of polyketone called polyarylalkylketone through the coupling of diketones such as 1,4-diacetylbenzene and diols such as 1,4-benzenedimethanol using a manganese pincer complex (Figure 13).^64^ Using this catalytic protocol, 12 new polyketones with molecular weights up to 899.1 kDa, exhibiting high decomposition temperatures and spherical morphology, were prepared. We also demonstrated the production of a polyketone using 1,4-benzendimethanol that was produced by the hydrogenative depolymerization of PET (polyethylene terepthalate) using Milstein’s RuPNN pincer catalyst. The mechanism is similar to that mentioned above for the synthesis of polyethylenimines and is based on the hydrogen-borrowing process (Figure 13B). The process starts with the dehydrogenation of diol to form an aldehyde (*initiation step*) that undergoes base-catalyzed aldol condensation with ketones to form an alkene intermediate (*propagation step*). Metal-catalyzed hydrogenation of alkenes leads to the formation of alkylated ketones.

#### 3.2.3 Synthesis of Polyesterethers Using Dehydrogenative and Hydrogen-Borrowing Polymerization

**Figure 14:**
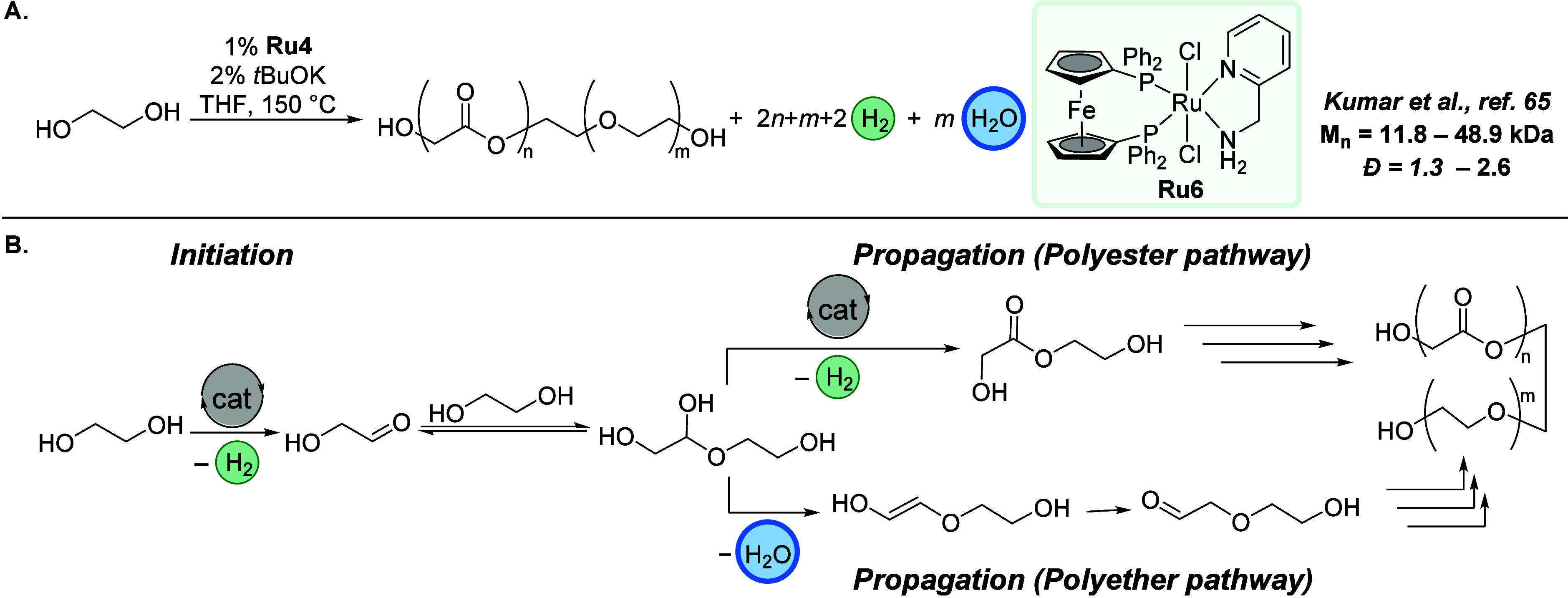
(A) Synthesis of polyesterethers via combined acceptorless dehydrogenation and hydrogen-borrowing polymerization. (B) Proposed mechanism for the formation of polyesterether.

Additionally, we introduced a new method for synthesizing aliphatic polyesterethers via bifunctional ruthenium-catalyzed dehydrogenative/dehydrative polymerization of ethylene glycol (Figure 14), with preliminary DFT computations indicating that both dehydration and dehydrogenation pathways are viable for forming polyesterethers.^65^ A notable conversion of 89% was achieved, producing a polyesterether with a molecular weight of 34 447 Da and an ester/ether ratio of 2.3:1. Similar polymerization was achieved with propylene glycol and glycerol (sourced from renewable feedstock), which also contain 1,2-diol units. The formation of polyester from acceptorless dehydrogenative coupling of ethylene glycol using a ruthenium acridine-based catalyst was also reported by Milstein.^66^ A mechanism for the formation of polyester and polyether is shown in Figure 14B. The dehydrogenation of a hemiacetal intermediate can lead to the formation of polyester, whereas the formation of a polyether occurs via dehydration followed by subsequent tautomerism.

Although the demonstration of the proof-of-concept of hydrogen-borrowing polymerization is promising from the perspective of using safer/renewable feedstock, there are certain challenges ahead of the road to scale up/commercialization. For example, (a) the formed polymers often have some unsaturation coming from the presence of imine or alkene depending on the reaction, as achieving 100% hydrogenation in the polymer chain is challenging. (b) As per the borrowing hydrogen process, the reaction needs to be done in a sealed flask, which means that the size of flask needs to be taken into account while optimizing the reaction conditions as this would influence the hydrogen pressure of the system, affecting the dehydrogenation/hydrogenation equilibrium. This makes the scaling up of the process complicated. (c) Currently, these processes have been demonstrated with a TON of 100, which would need significant improvement prior to any consideration of commercialization. This would require understanding of catalyst deactivation pathways that could assist in the development of more active catalysts as well as efficient catalyst recycling.

## 4 Synthesis of Main Group Polymers

**Figure 15:**
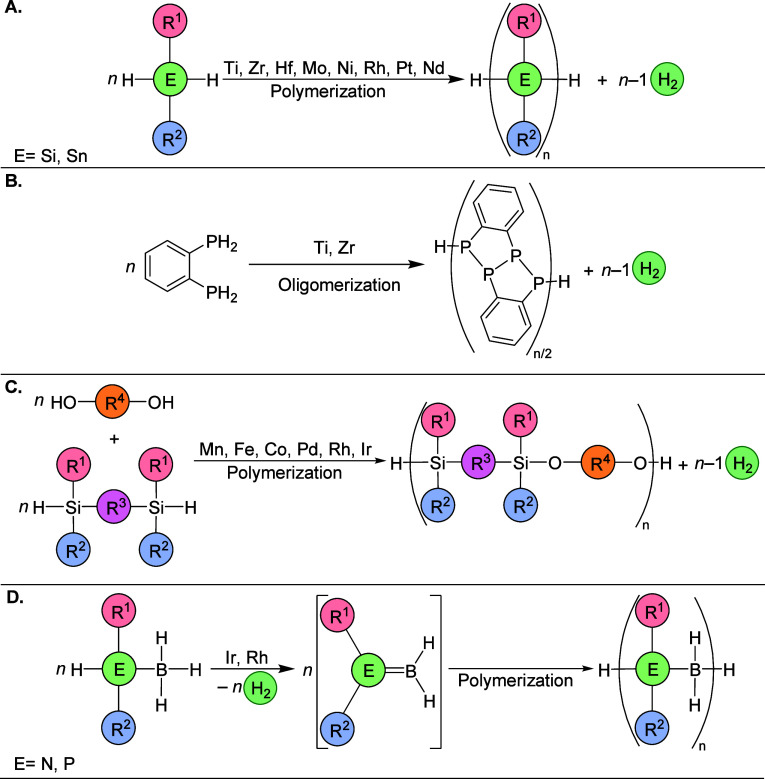
(A) Dehydrocoupling of silanes and stannanes. (B) Dehydrocoupling of phosphines. (C) Dehydrogenative synthesis of poly(siloxanes) and poly(silyl ethers). (D) Dehydrogenative polymerization of phosphineboranes and aminoboranes.

Polysilanes and polystanannes with only Si–Si and Sn–Sn bonds in the polymer skeleton, respectively, are the closest relatives to polyolefins made of single C–C bonds in their backbone. In contrast to polyolefins, the former can be synthesized via dehydrogenative polymerization of silanes and stannanes with only H_2_ as the byproduct (Figure 15A). The first report of polysilane synthesis by dehydrocoupling was published in 1973 by Ojima et al. using Wilkinson’s (Ph_3_P)_3_RhCl catalyst.^67^ Harrod and colleagues later developed an effective method using group IV metallocenes at room temperature.^68−70^ This method is best suited for monoorganosilanes, particularly arylsilanes, with CH_3_SiH_3_ being an exception. The highest molecular weight obtained with the Cp_2_ZrMe_2_ catalyst was *M*_n_ = 17.2 kDa (*Đ* = 2.0) for poly(3-trifluoromethylphenylsilane).^71^

Catalysts based on other transition metals apart from group IV were also explored, and it was found that the dehydropolymerization of primary and secondary silanes can be catalyzed by complexes of Mo, Rh, Ni, Pt, and Pd.^72^ Notably, using Pt(COD)_2_ as a catalyst, Tanaka and colleagues have managed to synthesize a polymer of relatively high molecular weight (*M*_n_ = 6.8–25 kDa) from a secondary silane, *n*HexMeSiH_2_.^73^ The mechanism proposed by Tilley for dehydrocoupling involves the activation of the Si–H bond by the activated catalyst containing an L_*n*_M–H bond via σ-bond metathesis. Simultaneous cleavage of Si–H and M–H bonds leads to the concomitant formation of the Si–M bond and evolution of H_2_. The species L_*n*_M-Si(H)RR′ interact with another molecule of silane to cleave another Si–H bond, along with the formation of Si–Si bond and regeneration of the active catalyst.^74^ Polysilanes obtained via dehydropolymerization have low molecular weights of *M*_n_ < 25 kDa,^75^ but they are an interesting class of polymers that contain the reactive Si–H groups in the chain.^76^ This provides opportunities for elaborating these polymers into other types by means of reactions like hydrosilylation. The exact reasons for the low molecular weights in the dehydrogenative polymerization of silanes remain unclear, but several factors likely contribute. These include the catalyst’s ability to cleave Si–Si bonds, leading to oligomeric or cyclic products, and the increasing viscosity of the reaction mixture, which may hinder catalyst access to growing polymer chains. Interestingly, dilution of the reaction mixture does not significantly improve molecular weights, suggesting additional complexities in the process that are not yet fully understood.^77^

Meanwhile, the most popular method for polysilane synthesis is Wurtz-type dehalogenative coupling of dichlorosilane. It is related to the fact that the most popular poly(silane) for applications in hybrid polymer synthesis,^78^ photoresist materials,^79^ electronic devices,^80^ photovoltaics,^81^ and semiconductor materials^82^ is polymethylphenylsilane, whose synthesis is well-established and produces a polymer of high molecular weight of around *M*_n_ = 100–500 kDa and narrow polydispersity.^75^ It is clear that new catalysts for dehydrocoupling that can deliver a polymer with high molecular weight and bulky substituents on silicon (e.g., secondary silanes with aryl substituents) would benefit the field. Currently, dehydrocoupling is mostly used for the modular synthesis of specially designed poly(silanes).^83,84^

Other polymers in this direction that have received attention are polystannanes which are primarily synthesized via catalytic dehydrocoupling of secondary alkyl or aryl tin dihydrides (Figure 15A), a method pioneered by Tilley in 1995.^85^ This process, carried out under mild conditions, produces a mix of cyclic and linear polymers with *M*_n_ up to 66 500 Da. While polystannanes are more challenging to prepare and handle due to lower Sn–Sn bond dissociation energies (190 kJ mol^–1^ vs 222 kJ mol^–1^ for Si–Si) and increased susceptibility to nucleophilic attack and photodegradation,^86^ several high-molecular-weight examples with narrow polydispersities have been reported. Both early (Ti, Zr, Hf)^85,87−89^ and late (Rh, Pt)^90−93^ transition metal complexes have successfully catalyzed the dehydropolymerization of primary and secondary stannanes. This method is particularly effective for poly(dialkylstannane) synthesis, with polymers from di-*n*-octylstannane reaching *M*_n_ = 97 kDa (*Đ* = 1.2),^91^ while alternative methods are preferred for aryl-substituted stannanes.^94^ For a comprehensive review on polystannane synthesis, refer to D. Foucher’s work.^95^ Mechanistic studies are scarce, but Tilley and Neale have managed to indicate that the dehydropolymerization of Mes_2_SnH_2_ (Mes = 2,4,6-trimethylphenyl) does not proceed via a σ-bond metathesis mechanism, as is the case for primary silanes, but more likely through α-H elimination.^96^ The dehydropolymerization in this case occurs via a chain growth polymerization mechanism that likely proceeds by the elimination of stannylene, Mes_2_Sn, followed by Sn–Sn bond formation from the insertion of the stannylene into the Sn–H bond of Mes_2_SnH_2_.

Catenated stannanes are unique main group polymers with intriguing optoelectronic and chemical properties due to their electronically delocalized metalloid backbones. While high-molecular-weight polystannanes have been synthesized, their sensitivity to moisture and light has limited testing and applications. The research in this area should focus on developing stable, processable polymers for use as one-dimensional polymeric wires, nonlinear optical materials, and semiconductors.^95^

Further in the direction of the synthesis of main group polymers via dehydrogenative pathways, there are reports that group IV metallocenes can catalyze the dehydrocoupling of primary phosphines, giving cyclic and linear oligomers^97−99^ (Figure 15B). It is hypothesized that the formation of the P–P bond occurs according to a σ-bond metathesis mechanism such as that in polysilane synthesis.^98^ Although the dehydrocoupling of substituted diphosphines and the formation of phosphorus–heteroatom bonds have been reported,^100−104^ this method has not evolved into the synthesis of high-molecular-weight poly(phosphines).

Other interesting polymers this direction are polysiloxanes. They have an annual industrial production exceeding 7 million tons^105^ and are primarily synthesized through the reaction of dichlorosilanes with water. For high-molecular-weight polymers, ring-opening polymerization (ROP) of cyclic oligosiloxanes (*x* = 3 and 4) using cationic or anionic initiation methods is preferred.^106^ However, dehydrogenative polymerization has emerged as an alternative method for specialized polymer synthesis (Figure 15C). For instance, rhodium(I)-catalyzed dehydrocoupling has been employed to copolymerize two optically active siloxane monomers, resulting in highly syndiotactic polysiloxane (*M*_n_ = 2400 g mol^–1^, *Đ* = 1.7, syndiotactic: 60%, heterotactic: 32%, isotactic: 8%).^107^ Despite this achievement, the physical properties of the syndiotactic polymer showed minimal differences when compared with those of its atactic counterpart.

Poly(silyl ethers), which are hybrids of organic polymers and siloxanes, offer diverse applications across industries. These versatile materials have been utilized to enhance polyether solubility in CO_2_,^108^ improve polymer membranes,^109^ and create stimuli-sensitive materials.^110^ Moreover, they show promise in space technology,^111^ microelectronics,^112^ and pharmaceuticals.^113^ Despite their potential, efficient synthesis methods remain elusive due to Si–O–C bond instability. Traditional approaches using dichlorosilanes and diols often yield cyclic compounds or low-molecular-weight oligomers.^114^ While high-molecular-weight polymers can be achieved using diphenoxy- and dianilinosilanes, these methods require extreme temperatures.^111,115,116^ Interestingly, dehydrogenative coupling of hydrosilanes with O-nucleophiles has emerged as a promising alternative (Figure 15C). This method offers improved chemoselectivity, efficiency, and scope while reducing waste and using milder conditions.^117^ Additionally, poly(silyl ethers) can now be synthesized from renewable resources.^118−120^ A wide range of catalysts have been employed for poly(silyl ether) synthesis, including main group elements,^121^ as well as transition metals such as Mn,^118,122,123^ Ir,^123^ Co,^124^ Pt,^125^ Fe,^126−128^ and Pd and Rh.^129,130^ This diverse array of catalytic options paves the way for further advancements in poly(silyl ether) synthesis and applications. Despite all the success that was achieved in the synthesis of poly(silyl ethers), the highest molecular weight obtained by the dehydrocoupling approach is still quite low, and the polymers are not stable in basic and acidic media. Overall, this precludes a mass application of the poly(silyl ethers), which have only been synthesized and tested in the laboratory so far.

In recent years, the field of main group polymers has witnessed exciting developments through the introduction of polyaminoboranes and polyphosphinoboranes (Figure 15D). These innovative materials serve as inorganic counterparts to traditional polyolefins, featuring B–N or B–P units in their main chain instead of C–C bonds.^131^ Despite their potential, these polymers remain largely unexplored compared to their organic analogues.^86,132^ Polyphosphinoboranes, in particular, have had a long and challenging journey toward synthesis. Initial attempts to create polymers with alternating phosphorus and boron atoms date back to the 1950s and 1960s, driven by the promise of high-temperature stability and flame retardancy.^133^ A significant breakthrough came in 1999 when Manners and colleagues discovered a rhodium(I)-catalyzed dehydrogenation method.^134^ This paved the way for further developments in transition-metal-catalyzed dehydrogenative synthesis of polyphosphinoboranes.^135−138^ The process has also been demonstrated using metal-free catalysts, which offer advantages such as higher molecular weights and broader applicability.^139,140^

Turning our attention to polyaminoboranes, while noncatalytic routes exist,^141,142^ metal-catalyzed approaches currently offer superior control over the polymerization process.^131,143^ The field has progressed rapidly since the first Ir-catalyzed dehydropolymerization of primary amine-boranes in 2008,^144^ with numerous catalyst systems now available.^143^ Notably, Weller developed a highly efficient Rh-based catalyst system, producing well-defined polymers with impressive molecular weights.^145^ The mechanism of dehydropolymerization is generally accepted to be a cascade-like polymerization,^146^ although alternative step-growth-like mechanisms have been proposed.^147,148^ Polyaminoboranes show great promise as precursors to hexagonal boron nitride (h-BN), a material with exceptional electronic, mechanical, and chemical properties.^149,150^ However, the challenge of separating the transition metal catalyst from the final product remains a critical area for improvement.^151,152^

The area of main group polymers also suffers from the challenges of recycling. Interestingly, recent research has shed light on the depolymerization of polyaminoboranes. Manners and colleagues demonstrated that strongly nucleophilic *N*-heterocyclic carbenes can promote the depolymerization of [H_2_BNMeH_2_]_*n*_ to form cyclic *N*,*N*,*N*-trimethylcyclotriborazane.^153^ Furthermore, Weller showed that *N*-methylpolyaminoborane can be depolymerized using catalytic amounts of sodium hexamethyldisilazide.^154^ These findings open up new possibilities for the recycling and modification of these innovative polymers, further expanding their potential applications in materials science.

## 5 Conclusions

As described above, the approach of dehydrogenative polymerization has been utilized to make various organic polymers, including polyheteroarenes, polyesters, polyamides, polyureas, polyureaurethanes, polyesterethers, polyethylenimines, and polyketones, as well as inorganic polymers such as polysilanes, polyaminoboranes, and polyphosphinoboranes. These are promising methodologies that allow access to new materials, avoid waste generation (except for oxidative dehydrogenative polymerization), and provide new routes to make renewable and recyclable polymers in a few cases. Recently, through the development of new methodologies to make several organic polymers, e.g., for the synthesis of polyureas, polyureaurethanes, polyketones, poly(ester ethers), and polyethylenimines, this class of polymerization has opened up new avenues. The approach of dehydrogenative polymerization to make such organic polymers is also attractive from a sustainability perspective, as this allows access to various renewable feedstock (e.g., renewable diols and diamines). Furthermore, the approach of catalytic hydrogenation has been utilized for the depolymerization of various polymers/plastics such as polyesters, nylons, polycarbonates, polyurethanes, and polyureas.^21^ Thus, the development of efficient methods to make polymers using the dehydrogenative polymerization process will be useful to the circular economy. However, none of these processes are close to being commercialized, and there are various challenges associated with each of the methodologies as discussed above. A lot of them come down to achieving the desirable physical and mechanical properties of the formed polymers as well as the cost of the process, which is directly linked with the activity and recyclability of the catalyst. Based on our studies, we identify the following needs for development in this area: (a) Synthesis of regioregular polyheteroaromatics of high molecular weight (e.g., > 10 000 Da) using the oxidative dehydrogenative approach will be useful, as such examples are limited and they are likely to meet the demands of material properties. (b) The synthesis of polyheteroaromatics is achieved using an oxidative dehydrogenation process starting from arenes feedstocks as described above. These processes also produce significant waste; therefore, demonstration of the synthesis of polyheteroaromatics using an acceptorless dehydrogenative route will be a breakthrough. Although dehydrogenative coupling of arenes using a thermal process is not known, such processes have been reported using photo/redox catalysis for small molecules.^155^ In terms of thermal catalysis, dehydrogenative coupling of alkenes has been reported using iridium pincer catalysts,^156^ and expansion of such processes to achieve the dehydrogenative coupling of arenes would allow the synthesis of polyheteroarenes using acceptorless dehydrogenative polymerization to be achieved. (c) Acceptorless dehydrogenative coupling to make polycarbonates has not yet been reported. A possible route for the dehydrogenative synthesis of polycarbonates could be the dehydrogenative coupling of methanol with diols. This would present a greener alternative to the conventional synthesis of polycarbonates that involves the coupling of diols with phosgene gas, which is highly toxic. Significant advances have been made in the synthesis of polycarbonates from copolymerization of epoxide and CO_2_.^157^ However, the use of diols as a feedstock can present advantages in comparison to epoxides in terms of cost and substrate scope. Similarly, the synthesis of polyurethanes via acceptorless dehydrogenative coupling of diols, diamines, and methanol will be greener than the current industrial route that involves diisocyanate feedstock. Furthermore, the reverse reactions, i.e., hydrogenative depolymerization of polycarbonates to diols and methanol as well as hydrogenative depolymerization of polyurethanes to diamines, diols, and methanol, have been reported a few times.^21^ This means that the development of efficient methods for the polymerization process will close the loop for the production/recycling of polycarbonates and polyurethanes. (d) The dehydrogenative polymerization processes have only been demonstrated using homogeneous organometallic catalysts, and in most cases turnover numbers are less than 100. In general, there is a need to develop more active homogeneous catalysts (TON > 10,000) or develop an efficient method for recycling such catalysts. Considering that most of the polymer precipitates out from the reaction mixture and it is likely that the active species remains soluble, catalyst recycling can be easily achieved by filtration if the catalyst remains stable. Furthermore, the use of heterogeneous catalysts or photocatalysts has also not been employed for these processes. Considering that both thermal heterogeneous catalysts^158,159^ and photoredox catalysis^155^ have been reported for the dehydrogenation of alcohols, there are ample opportunities to evaluate these catalysts in dehydrogenative polymerization processes. (e) The area of dehydrogenative polymerization, especially for organic polymers, is still underexplored, and as such none of these processes have been scaled-up, which would be needed for polymer processing and understanding of rheology properties. This is again linked with catalysis development in terms of developing highly active or recyclable catalysts. (f) The dehydrogenative coupling approach was demonstrated to make catenated polysilanes and polystannanes with good molecular weights (*M*_n_) around 10 kDa, but the available substrate scope is still limited to primary silanes and unhindered stannanes. Hence, a highly active catalyst that is capable of making polymers with *M*_n_ of more than 100 kDa in a controllable fashion is required for the dehydrogenation to be a method of choice over classic Wurtz-type dehalogenation. Moreover, additional requirements would be developing dehydrogenative methods to make polysilanes and polystannanes containing bulky substituents that could make these polymers more stable and robust. (g) Low catalytic activity and polymer molecular weight also hinder the application of dehydrogenative polymerization for the synthesis of poly(silyl ethers) and poly(siloxanes); therefore, further development of efficient catalytic processes to make high-molecular-weight poly(silyl ethers) and poly(siloxanes) would be beneficial. (h) As mentioned above the synthesis of polyaminoboranes has been demonstrated using a fairly active catalyst (e.g., TON of 10 000), and the polymer processing as well as mechanical properties have also been studied.^160^ Further testing of these materials for desirable applications, such as boron-based pre-ceramic materials, as well as developing methods of chemical recycling will be future steps toward their commercial application.
